# Splenic switch-off as a novel marker for adenosine response in nitrogen-13 ammonia PET myocardial perfusion imaging: Cross-validation against CMR using a hybrid PET/MR device

**DOI:** 10.1007/s12350-020-02448-y

**Published:** 2020-12-22

**Authors:** Adam Bakula, Dimitri Patriki, Elia von Felten, Georgios Benetos, Aleksandra Sustar, Dominik C. Benz, Muriel Wiedemann-Buser, Valerie Treyer, Aju P. Pazhenkottil, Christoph Gräni, Catherine Gebhard, Philipp A. Kaufmann, Ronny R. Buechel, Tobias A. Fuchs

**Affiliations:** Department of Nuclear Medicine, Cardiac Imaging, University Hospital Zurich, Ramistrasse 100, 8091 Zurich, Switzerland

**Keywords:** Hybrid imaging, MPI, Vasodilators, Myocardial blood flow, PET, MRI

## Abstract

**Background:**

No methodology is available to distinguish truly reduced myocardial flow reserve (MFR) in positron emission tomography myocardial perfusion imaging (PET MPI) from seemingly impaired MFR due to inadequate adenosine response. The adenosine-induced splenic switch-off (SSO) sign has been proposed as a potential marker for adequate adenosine response in cardiac magnetic resonance (CMR). We assessed the feasibility of detecting SSO in nitrogen-13 ammonia PET MPI using SSO in CMR as the standard of reference.

**Methods and Results:**

Fifty patients underwent simultaneous CMR and PET MPI on a hybrid PET/MR device with co-injection of a gadolinium-based contrast agent and nitrogen-13 ammonia during rest and adenosine-induced stress. In CMR, SSO was assessed visually (positive vs negative SSO) and quantitatively by calculating the ratio of the peak signal intensity of the spleen during stress over rest (SIR). In PET MPI, the splenic signal activity ratio (SAR) was calculated as the maximal standard uptake value of the spleen during stress over rest. The median SIR was significantly lower in patients with positive versus negative SSO in CMR (0.57 [IQR 0.49 to 0.62] vs 0.89 [IQR 0.76 to 0.98]; *P* < .001). Similarly, median SAR in PET MPI was significantly lower in patients with positive versus negative SSO (0.40 [IQR 0.32 to 0.45] vs 0.80 [IQR 0.47 to 0.98]; *P* < .001).

**Conclusion:**

Similarly to CMR, SSO can be detected in nitrogen-13 ammonia PET MPI. This might help distinguish adenosine non-responders from patients with truly impaired MFR due to microvascular dysfunction or multivessel coronary artery disease.

## Introduction

Positron emission tomography myocardial perfusion imaging (PET MPI) is a robust and excellent tool for quantitative and semi-quantitative assessment of myocardial blood flow.^1^ Besides physical exercise or dobutamine stress, pharmacological vasodilators such as adenosine, regadenoson or dipyridamole are commonly used to induce coronary hyperaemia. However, adequate patient response to the latter is crucial to detect ischemia or reduced myocardial flow reserve (MFR), and conversely, an inadequate response might result in a false-negative result, decreased extent of ischemia or seemingly reduced MFR. Rates of up to 5% to 10% of false-negative MPI examinations have been reported,^2^,^3^ and up to one-third of these may be attributed to inadequate stress.^4^ Haemodynamic parameters such as an increase in heart rate or a drop in systolic blood pressure are commonly used to assess the response to vasodilators, however, whether this reflects true coronary response remains doubtful.^5^,^6^ Recently, for patients undergoing stress cardiac magnetic resonance (CMR), the splenic switch-off (SSO) sign, which is the visually assessed decrease in splenic enhancement during adenosine stress, has been proposed to be a marker for haemodynamic response. Its validity as a marker for adenosine response is based on the assumption that while adenosine receptor stimulation results in splanchnic vasodilation, the splenic circulation is not affected by this mechanism, potentially even showing an opposite reaction in the form of vasoconstriction.^7^,^8^ The utility of SSO for identifying inadequate adenosine response in CMR was first described in the CE-MARC study cohort, where significantly more false-negative than true negative exams failed to show the sign.^9^ A subsequent study in a real-world population also showed an increased prevalence of SSO in true positive as compared to false-negative CMR exams, although not reaching statistical significance.^10^

In the present study, we test the hypothesis that similarly to CMR, SSO can be assessed in nitrogen-13 ammonia PET MPI. To this aim, we assessed patients undergoing simultaneous CMR and PET MPI with adenosine-induced stress on a hybrid PET/MR device with co-injection of a gadolinium-based contrast agent (GBCA) and nitrogen-13 ammonia using SSO in CMR as the standard of reference.

## Methods

### Study design and population

Data of this prospective single-centre study were derived from ongoing PET/MR projects. We assessed patients who underwent cardiac PET/MR for evaluation of coronary artery disease (CAD) or myocarditis and obtained written informed consent from all patients. The study protocol was approved by the local ethics committee (KEK-ZH-Nr. 2014-0187 and BASEC-Nr. 2018-00170). Patients younger than 18 years, and patients with contraindications against CMR (e.g. non-CMR-compatible implanted cardiac devices, claustrophobia), GBCA (known allergy, severe renal impairment), adenosine (e.g. asthma, atrioventricular block) or PET (e.g. pregnancy or breastfeeding) were excluded. The patients were instructed to abstain from caffeine intake for at least 12 hours prior to the examination. This work was supported by a grant from the Swiss National Science Foundation (SNSF, Project No. 175640).

### Hybrid PET/MR acquisition protocol

A hybrid PET/MR scanner incorporating a 3 Tesla MR and a PET scanner with time-of-flight (TOF) (Signa PET/MR, GE Healthcare, Waukesha, WI, USA) was used for the simultaneous acquisition of PET and CMR datasets. For the stress protocol, a weight-adapted adenosine infusion (140 μg·kg^−1^·min^−1^) with a total length of 6 minutes was used. Three minutes into adenosine stress, a body mass index-adapted dose of nitrogen-13 ammonia (i.e. 200 to 600 megabecquerels [MBq], 5.41 to 16.22 millicurie [mCi]) and a weight-adapted dose of a gadolinium-based contrast agent (Gadovist, Bayer AG, Zurich, Switzerland; 0.1 mmol·kg^−1^) were injected.

PET data were acquired in list mode and reconstructed as a static, dynamic (7 min divided into 21 frames: 9 × 10, 6 × 15, 3 × 20, 2 × 30, and 1 × 120 seconds), and an ECG-gated dataset (10 min) using TOF reconstruction with VUE Point FX (2 iterations and 16 subsets) and a 5-mm Hanning filter. Standard DIXON-based maps were used for attenuation correction.^11^ The resting PET images were acquired at least 15 min after the stress acquisition using an identical imaging protocol, after the injection of approximately double the stress dose of nitrogen-13 ammonia.

For CMR MPI, three short-axis slices of the left ventricle (basal, midventricular and apical; 10-mm slice thickness) were acquired per cardiac cycle during breath-hold. T1-weighted fast gradient echo sequences with short TR and TE (TR 3.3 ms TE 1.2 ms, flip angle 20°) were used after a 90° non-selective saturation preparation pulse with a saturation delay time of 100 ms and a typical acquired voxel size of 2.9 × 2.9 mm^2^ and a matrix size of 128x128 (frequency × phase). To reduce the slice acquisition time, a parallel imaging acceleration factor of 2 (Asset) was used. The resting CMR images were acquired at least 15 minutes after the stress acquisition using an identical imaging protocol and the same weight-adapted dose of a gadolinium-based contrast agent.

### MR analysis

Qualitatively, positive SSO in CMR was defined as a visually perceivable lower splenic enhancement on stress compared to rest first-pass CMR images, as previously reported.^9^ The visual analysis was performed by two independent readers in a blinded fashion. In the case of disagreement, consensus with a third independent reader was sought. Greyscale values were normalized and identical values were applied to stress and rest images for better comparability.

**Figure 1:**
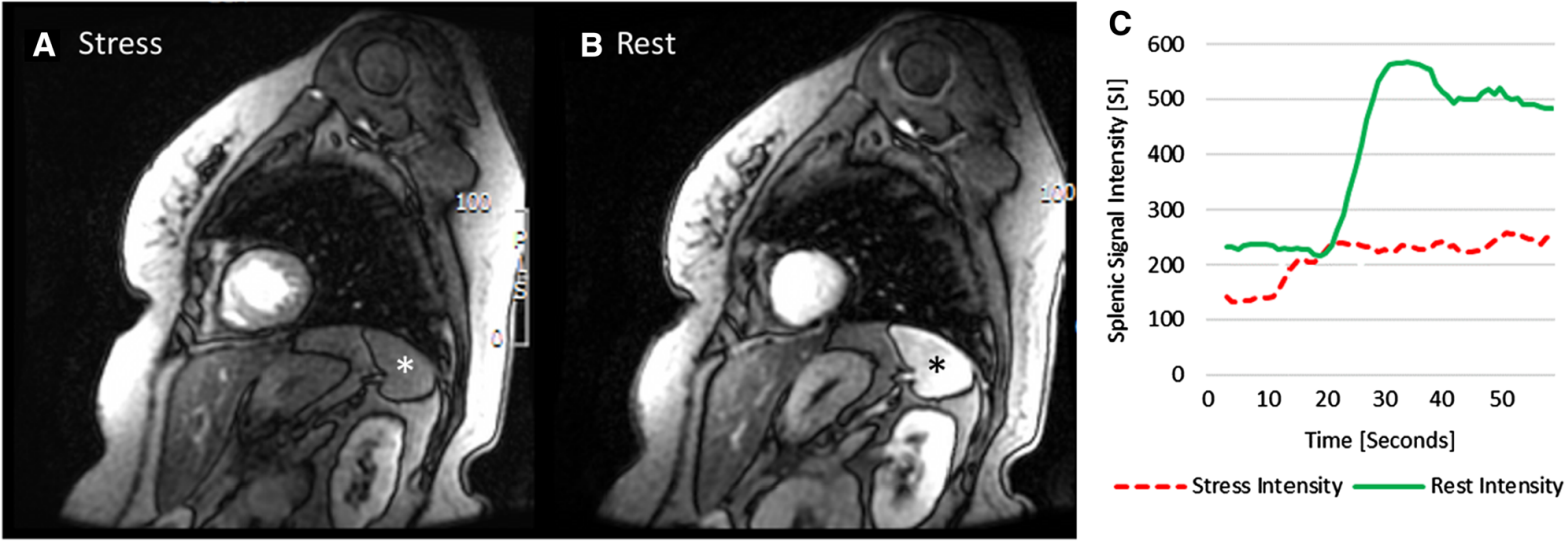
CMR short-axis first-pass perfusion images in a patient undergoing adenosine stress PET/MR with positive SSO (*) defined as visually perceivable lower splenic enhancement on first-pass stress (**A**) compared to rest (**B**) perfusion and the corresponding splenic signal intensity curves, showing an SIR of 0.37 (**C**)

For quantitative SSO analysis, regions of interest (ROIs) were drawn in the spleen on stress and rest images to generate time–intensity curves using commercially available software (CMR 42 Version 5.9.4, Circle Cardiovascular Imaging, Calgary, Alberta, Canada). Corrected peak signal intensities of the spleen were calculated for stress and rest by subtracting baseline intensity (pre-contrast) from peak intensity after first-pass perfusion. Finally, the splenic intensity ratio (SIR) was calculated by dividing the baseline-corrected peak splenic intensity during stress by the baseline-corrected peak splenic intensity at rest (SIR = (splenic intensity_Stress_−splenic intensity_Baseline_)/(splenic intensity_Rest_−splenic intensity_Baseline_)), (Figure 1).

### PET analysis

**Figure 2:**
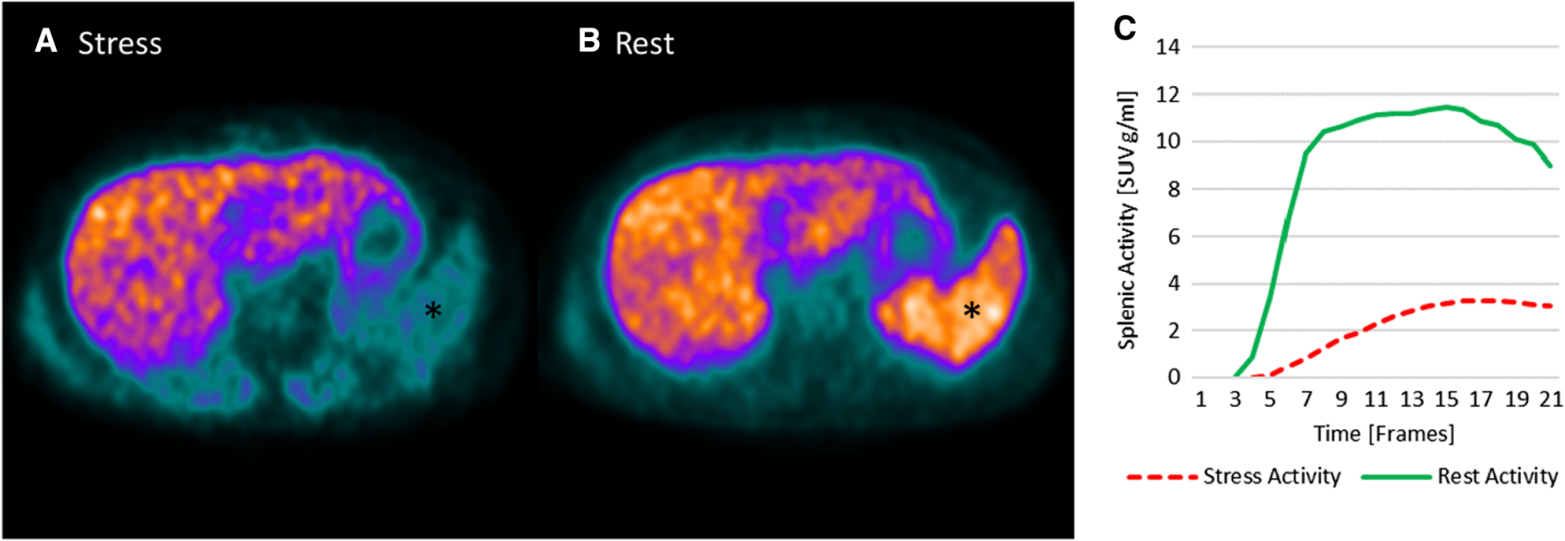
Nitrogen-13 ammonia PET in a patient undergoing adenosine stress PET/MR. A transaxial slice through the spleen is shown. There is lower tracer activity of the spleen (*) during stress (**A**) as compared to rest (**B**). Similarly, a quantitative assessment by splenic time activity curves, showing a SAR of 0.29 (positive SAR) (**C**)

To quantitatively assess SSO in PET, the activity values in the stress and rest datasets were converted into standardized uptake values (SUV) using the administered tracer dose, tracer half-life and time of administration and recorded using commercially available software (PMOD Software Package Version 3.805, PMOD Technologies LLC, Zürich, Switzerland). Volumes of interest were placed in the spleen (10-mm radius sphere) and liver (20-mm radius sphere) to generate time–activity curves from the dynamic PET datasets. The splenic activity ratio (SAR) was calculated as the peak splenic activity during stress over the peak splenic activity at rest (SAR = splenic activity_Stress_/splenic activity_Rest_), (Figure 2). The spleen-to-liver activity ratio during stress (SLR_Stress_) was calculated by dividing the peak splenic activity during stress by the peak liver activity during stress (SLR_Stress_ = splenic activity_Stress_/liver activity_Stress_). Similarly, the spleen-to-liver activity ratio during rest (SLR_Rest_) was calculated by dividing the peak splenic activity during rest by the peak liver activity during rest (SLR_Rest_ = splenic activity_Rest_/liver activity_Rest_). The stress-to-rest spleen-to-liver activity ratio (SLR_Stress/Rest_) was calculated by dividing the SLR_Stress_ by SLR_Rest_ (SLR_Stress/Rest_ = SLR_Stress_/SLR_Rest_). Quantitative myocardial blood flow (MBF) was obtained from stress and rest PET scans, and global MFR was calculated as the ratio of stress over rest MBF using commercially available software (CSI 2017.7 Cedars-Sinai Medical Center, Los Angeles, CA, USA). An MFR < 1.7 was defined as impaired flow reserve.^12^

### Statistical analysis

The continuous parameters were tested for normal distribution using the Kolmogorov–Smirnov test and are presented as mean ± SD for normally distributed variables and as median and interquartile range (IQR) for non-normally distributed variables. To compare groups, *T* Test was used for normal distributed parameters and Mann–Whitney-*U*-Test for non-normally distributed parameters. For categorical parameters, a Chi^2^-Test was used. Correlations were analysed using Spearman’s rho. Cut-off values for activity ratios predicting positive SSO in CMR were calculated using receiver operating characteristic (ROC) analysis. For statistical analysis, IBM SPSS Statistics version 22 (IBM Corporation, Armonk, NY, USA) was used.

## Results

All 50 patients successfully underwent adenosine stress PET/CMR. Detailed patient baseline characteristics are given in Table 1.

**Table 1:** Baseline characteristics, risk factors, medication, medical history and PET MPI results in patients with positive and negative SSO

	All patients (50)	Positive SSO (37)	Negative SSO (13)	*P*
Baseline characteristics				
Median age (years), *n* (IQR)	47 (25-62)	47 (28–58)	34 (21–69)	0.851
Male gender *n* (%)	39 (78)	28 (75.7)	11 (84.6)	0.503
Weight (kg), *n* (± SD)	79.6 (10.9)	80.4 (10.8)	77.3 (11.3)	0.379
Height (cm), *n* (± SD)	175 (9)	175 (9)	178 (8)	0.247
BMI (kg/m^2^), *n* (± SD)	25.9 (3.2)	26.4 (3.0)	24.4 (3.2)	0.53
Risk factors				
Diabetes mellitus, *n* (%)	1 (2)	1 (3)	0 (0)	0.549
Dyslipidaemia, *n* (%)	15 (30)	12 (32)	3 (23)	0.527
Hypertension, *n* (%)	13 (26)	11 (30)	2 (15)	0.31
Family history, *n* (%)	13 (26)	10 (27)	3 (23)	0.78
Smoking, *n* (%)	19 (38)	17 (46)	2 (15)	0.051
Medication				
Aspirin, *n* (%)	13 (26)	9 (24)	4 (31)	0.649
Beta blocker, *n* (%)	10 (20)	8 (22)	2 (15)	0.629
ACE inhibitor, *n* (%)	14 (28)	10 (27)	4 (31)	0.796
Statin, *n* (%)	15 (30)	10 (27)	5 (39)	0.439
Patient history				
CAD, *n* (%)	14 (28)	10 (27)	4 (31)	0.796
Myocardial infarction, *n* (%)	7 (14)	4 (11)	3 (23)	0.273
Stenting, *n* (%)	8 (16)	5 (14)	3 (23)	0.418
CABG, *n* (%)	4 (8)	1 (3)	3 (23)	**0.02**
Nitrogen-13 ammonia PET				
Stress nitrogen-13 ammonia dose, MBq/mCi (IQR)	264 (254–276)/7.14 (6.86–7.46)	267 (254–274)/7.22 (6.86–7.41)	258 (250–289)/6.97 (6.76–7.81)	0.595
Rest nitrogen-13 ammonia dose, MBq/mCi (IQR)	513 (434–533)/13.86 (11.73–14.41)	514 (453–537)/13.89 (12.24–14.51)	509 (410–524)/13.76 (11.08–14.16)	0.283
Stress MBF (ml·min^−1^·g^−1^) (± SD)	2.32 (0.72)	2.37 (0.62)	2.17 (0.95)	0.373
Rest MBF (ml·min^−1^·g^−1^) (IQR)	0.73 (0.6–0.86)	0.67 (0.58–0.83)	0.77 (0.71–0.88)	0.141
MFR, (± SD)	3.16 (0.93)	3.38 (0.86)	2.53 (0.84)	**0.003**

Bold values indicate *P* < 0.05

*SSO* splenic switch-off, *IQR* interquartile range, *SD* standard deviation, *BMI* body mass index, *ACE* angiotensine converting enzyme, CAD coronary artery disease, *CABG* coronary artery bypass graft, *MBF* myocardial blood flow, *MFR* myocardial flow reserve

**Figure 3:**
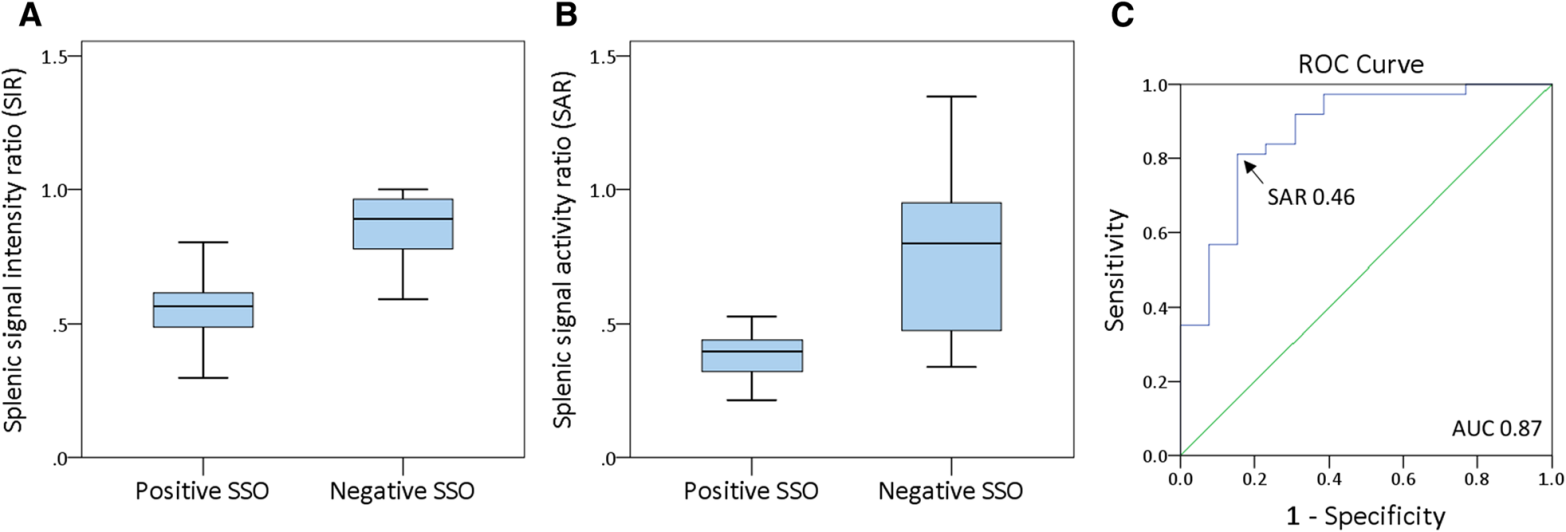
Median splenic SIR in CMR (**A**), median splenic SAR in PET (**B**) in patients with positive versus negative SSO and ROC analysis for splenic SAR to detect positive SSO in CMR (area under the curve of 0.87) (**C**)

**Figure 4:**
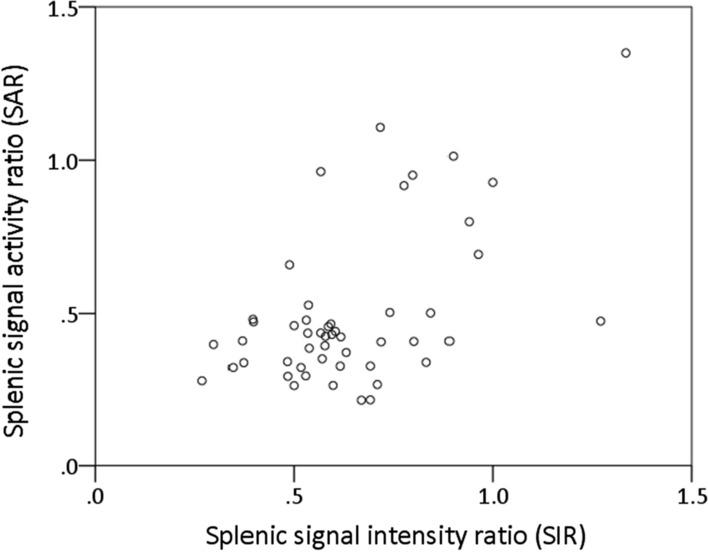
Correlation of SIR and SAR

Positive SSO in CMR was found in 37 patients (74%). SIR, as derived from CMR, was significantly lower in patients with positive versus negative SSO (0.57 [IQR 0.49 to 0.62] vs 0.89 [IQR 0.76 to 0.98]; *P* < .001; Figure 3a). Similarly, SAR, as derived from PET, was significantly lower in patients with positive versus negative SSO (0.4 IQR [0.32 to 0.45] vs 0.8 IQR [0.47 to 0.98]; *P* = .001; Figure 3b). ROC analysis revealed an area under the curve of 0.87 for SAR to detect positive SSO and a cut-off value of 0.46 *(*Figure 3c). Using this threshold, a sensitivity of 81%, specificity of 85%, PPV of 94% and NPV of 61% were calculated. SSO and SAR ≤ 0.46 showed moderate agreement (*κ *= 0.58, *P* < .001). SIR and SAR showed a weak, but statistically significant correlation (*σ* = 0.38, *P* = .007; Figure 4).

**Figure 5:**
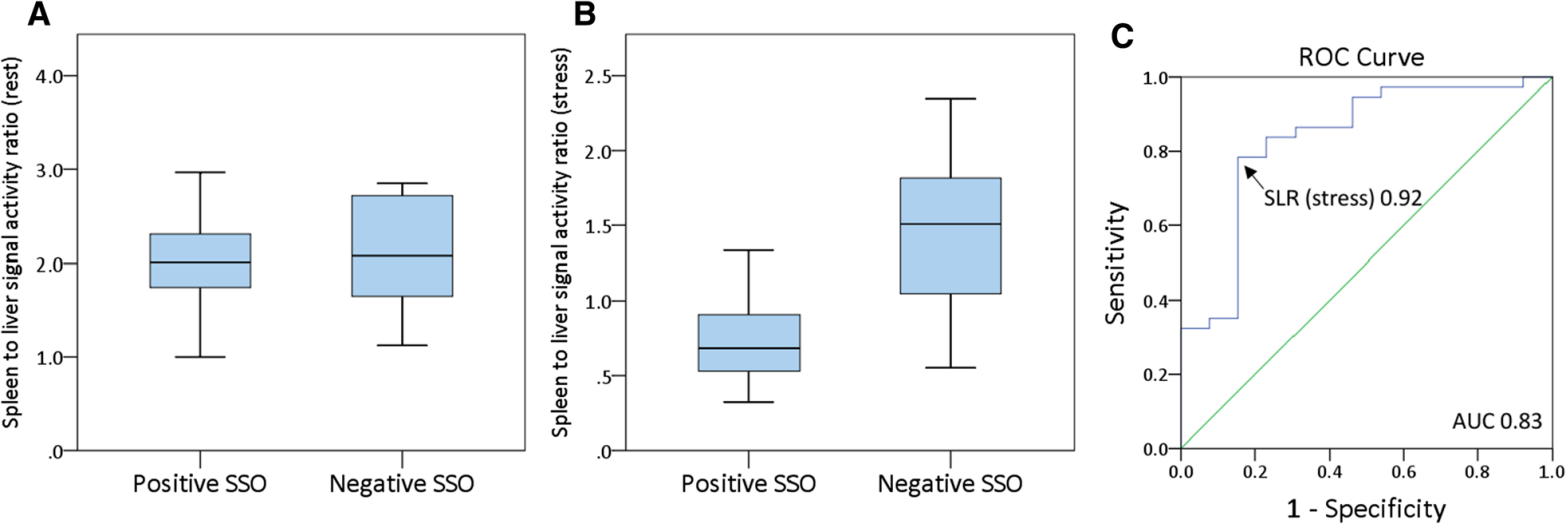
Median spleen-to-liver activity ratio at rest in PET (**A**), median spleen-to-liver activity ratio under stress in PET (**B**) in patients with positive versus negative SSO and ROC analysis for spleen-to-liver activity ratio under stress to detect positive SSO in CMR (area under the curve of 0.83) (**C**)

SLR_Stress_ was significantly lower than SLR_Rest_ (0.75 [IQR 0.55 to 1.15] vs. 2.05 [IQR 1.7 to 2.36]; *P* < .001). SLR_Rest_ did not differ significantly between patients with positive versus negative SSO (2.05 [± 0.49] vs. 2.12 [± 0.55]; *P* = .666; Figure 5a). SLR_Stress_ was significantly lower in patients with positive versus negative SSO (0.68 [IQR 0.51 to 0.91] vs. 1.5 [IQR 0.98 to 1.83]; *P* < .001; Figure 5b). ROC analysis for SLR_Stress_ to detect positive SSO showed an area under the curve of 0.83 and a cut-off value of 0.92 (Figure 5c). Using this threshold, a sensitivity of 78%, specificity of 85%, PPV of 94% and NPV of 58% were calculated. SSO and SLR_Stress_ < 0.92 showed moderate agreement (*κ *= 0.55, *P* < .001).

**Figure 6:**
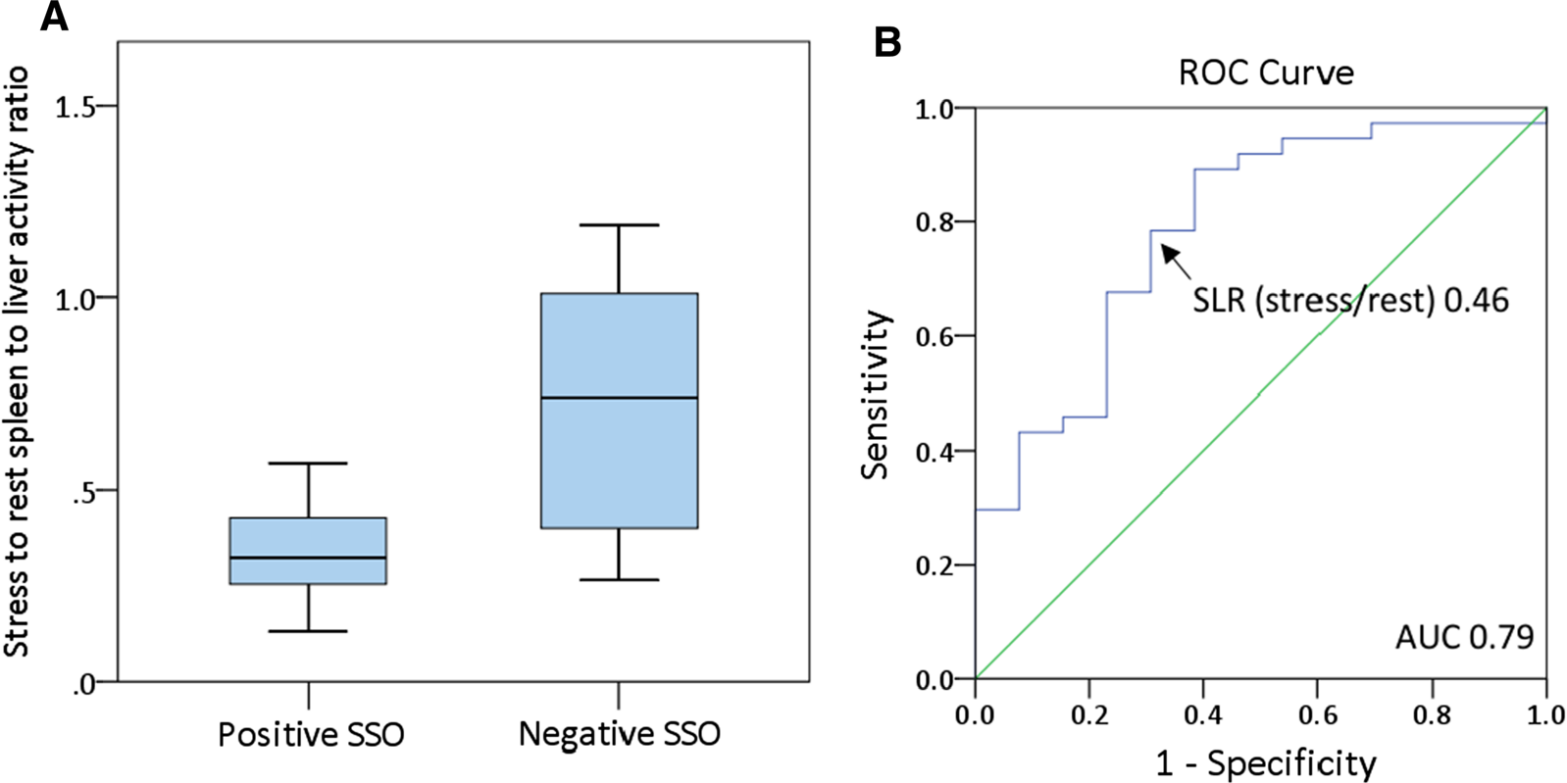
Median stress-to-rest spleen-to-liver activity ratio in PET in patients with positive versus negative SSO (**A**) and ROC analysis for stress-to-rest spleen-to-liver activity ratio to detect positive SSO in CMR (area under the curve of 0.79) (**B**)

SLR_Stress/Rest_ was significantly lower in patients with positive versus negative SSO (0.32 [IQR 0.25 to 0.44] vs. 0.74 [IQR 0.36 to 1.05]; *P* = .002; Figure 6a). The ROC analysis for SLR_Stress/Rest_ to detect positive SSO showed an area under the curve of 0.79 and a cut-off value of 0.46 (Figure 6b). Using this threshold, a sensitivity of 78%, specificity of 69%, PPV of 88% and NPV of 53% were calculated. SSO and SLR_Stress/Rest_ < 0.46 showed moderate agreement (*κ *= 0.43, *P* < .01).

Mean MFR was significantly higher in patients with positive vs negative SSO in CMR (3.38 ± 0.86 vs 2.53 ± 0.84; *t* = 3.09, df = 48, *P* = .003). Similarly, in patients with SAR ≤ 0.46 (positive SAR), the mean MFR was significantly higher than in patients with SAR above this threshold (3.43 ± 0.82 vs 2.69 ± 0.95; *t* = 2.87, df = 48, *P* = .006). In patients with SLR_Stress_ < 0.92, the mean MFR was significantly higher than in patients with SLR_Stress_ above this threshold (3.52 ± 0.82 vs 2.58 ± 0.81; *t* = 3.94, df = 48, *P* < .001). In patients with SLR_Stress/Rest_ < 0.46, the mean MFR was significantly higher than in patients with SLR_Stress/Rest_ above this threshold (3.41 ± 0.90 vs 2.69 ± 0.80; *t* = 2.77, df = 48, *P* = .008).

## Discussion

To the best of our knowledge, this is the first study demonstrating the feasibility of detecting and quantifying SSO in adenosine stress nitrogen-13 ammonia PET MPI. We established a novel indicator for the presence of SSO in nitrogen-13 ammonia PET: the SAR, which was calculated by dividing the peak splenic activity during stress over the peak splenic activity at rest similar to the SIR in CMR. A SAR of 0.46 was found to be a highly accurate threshold to differentiate positive from negative SSO, as depicted in CMR. It should be noted that the results relate to the prediction of the visual interpretation of SSO and not directly to the vasodilator efficacy. Nevertheless, the study provides evidence that PET-derived quantitation of spleen activity in the form of SAR provides similar information on the adequacy of adenosine response as the MR-derived SSO.

We have applied a comparable and easy measurable method for quantification of SAR using peak activity measurements, as shown for SIR in CMR.^9^,^10^ These quantification methods resulted in some overlap in results as compared to visual SSO in CMR. This could be due to the fact that both timing and intensity can influence the interpretation of visual splenic switch-off, as opposed to SIR and SAR, which are derived from single data points. In further studies, using coronary anatomy as the standard of reference, alternative methods of quantification, i.e. the integral of the splenic activity over time, or kinetic parameters could be investigated. In contrast to CMR, where the same dose of a gadolinium-based contrast agent is injected for stress and rest, different activities of the tracer are usually administrated for stress and rest examinations in nitrogen-13 ammonia PET. Therefore, it is crucial to convert activity values of the datasets into SUV, in order to enable splenic activity comparison. This can be done using PET scanners undergoing routine quality control including dose calibration and commercially available software. The nitrogen-13 ammonia dose used for SUV calculations in our study was corrected with residual dose measurements in case if manual injections, or an automatic dose drawing system was used, with no residual dose measurements. The residual dose in the patient line and intravenous cannula was not measured, also an approximated time of 1 minute between the dose measurement and injection was used. Since this approach was used in both, the stress and rest acquisitions, there was no systemic bias influencing the ratio of splenic activities. Also the doses individually were within the linear sensitivity spectrum of the scanner, therefore we can assume that this approach works correctly. Alternatively, organs like the liver can be used as a reference allowing the calculation of dose-independent ratios. Therefore, we additionally showed that the median spleen-to-liver activity ratio under stress was significantly lower in patients with visual SSO in CMR compared to patients without visual SSO.

**Fig. 7:**
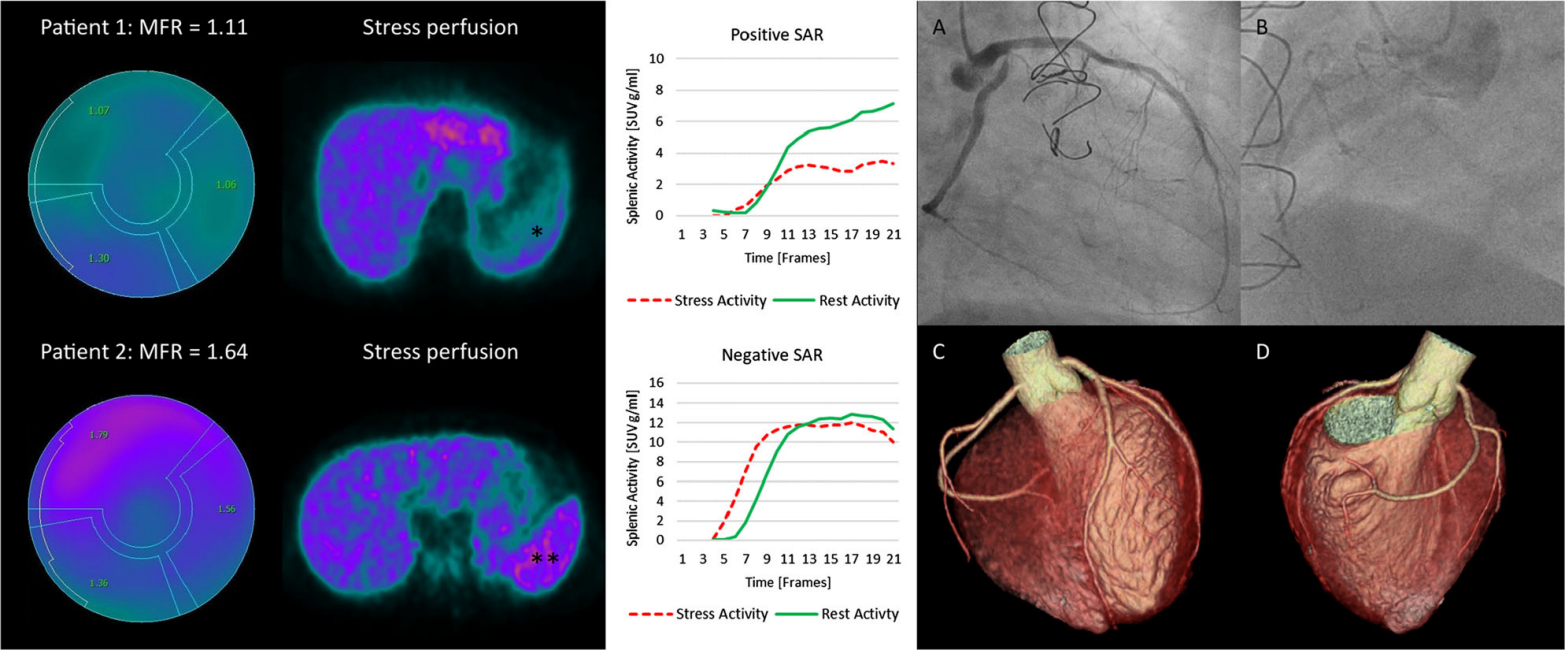
An example of two patients with impaired MFR. Invasive coronary angiography of patient #1 (top) with impaired MFR of 1.11 and a SAR of 0.41 (positive SSO: *) revealed severe CAD with a subtotal stenosis of the ostial left circumflex artery (**A**), occluded RCA (**B**) and subtotal stenosis of LIMA-LAD bypass graft, indicating truly reduced MFR. Coronary computed tomography (C,D) in patient #2 (bottom) with impaired MFR of 1.64 and a SAR of 0.93 (negative SSO: **) excluded obstructive CAD or calcifications, indicating failed adenosine response as the cause for the impaired MFR

Only two patients in our study had impaired MFR, which did not allow for statistical analysis of the differences in SAR, or SLR between patients with normal and impaired MFR in this population. In the two patients with impaired MFR, the threshold for SAR correctly differentiated a true positive from false positive: Invasive coronary angiography of one patient with an MFR of 1.11 and a SAR of 0.41 (i.e. positive SAR) revealed obstructive multivessel CAD. By contrast, in the second patient (21-year old, with no cardiovascular risk factors except light smoking) with impaired MFR of 1.64 but a SAR of 0.93 (i.e. negative SAR), coronary computed tomography showed normal coronary arteries, suggesting inadequate adenosine response as the reason for the false-positive PET finding (Figure 7). As SAR is a novel parameter, it has not yet been used in MPI PET. However, the clinical advantage of SAR might warrant its implementation, especially in patients with reduced MFR in whom an adenosine non-responder or inadequate response cannot be excluded. Taking into account that a considerable number of patients might be understressed,^2^–^4^ a novel marker in nitrogen-13 ammonia MPI PET would be more than welcome to differentiate truly from seemingly reduced MFR. This can help to further improve the already established substantial diagnostic and prognostic value of MFR in nitrogen-13 ammonia MPI PET.^13^

In our cohort, mean MFR in PET was significantly higher in patients with a splenic SAR ≤ 0.46 (i.e. positive SAR) as compared to patients with a SAR > 0.46 (i.e. negative SAR). This was also true for the other activity ratios in PET MPI: SLR_Stress_ and SLR_Stress/Rest_. The conclusion that this observation might be due to a more pronounced coronary adenosine response has to be drawn with caution as MFR can be reduced in patients with an adequate adenosine response due to severe CAD or microvascular dysfunction. Also, although SAR showed the best agreement with visual SSO in CMR, patients with SLR_Stress_ < 0.92 and SLR_Stress_ ≥ 0.92 showed the most significant differences in MFR, suggesting the best correlation with the haemodynamic response to vasodilator stress. All three activity ratios should be compared, when validating SSO in PET MPI against coronary anatomy.

There is growing evidence about the incremental value of SSO in stress CMR to predict adequate adenosine response,^9^,^10^,^14^,^15^ although there is also literature demonstrating that SSO in CMR failed to predict false-negative adenosine results after caffeine intake.^16^ Only one study examined this phenomenon in Rb-82 PET MPI, 17 despite the fact that initial observations about reduced splenic tracer activity during stress have been described in 1984.^18^ In line with the former CMR studies, Bami et al found an incremental prognostic value of a splenic response ratio in PET.^17^ However, this PET study did not aim at cross-validation versus a standard of reference, while the simultaneity of assessment of both markers through co-injection and use of a hybrid PET/MR device virtually excluded intrapatient variability of adenosine response in our study. Prognostic outcomes were not the subject of our study, as the population was not representative of a typical population of patients undergoing PET MPI.

Compared to CMR studies by Manisty et al (90%) 9 and Hoskins et al (89%), 10 positive SSO in our study was less frequent. Reasons for the slightly lower prevalence (74%) in our cohort might be the different doses of adenosine used in the studies. While we used a fixed adenosine infusion dose of 140 μg·kg^−1^·min^−1^, both other studies titrated adenosine up to 175 μg·kg^−1^·min^−1^. Indeed, current guidelines for nuclear cardiology procedures recommend a fixed infusion of 140 μg·kg^−1^·min^−1^ adenosine,^19^ supported by the fact that uptitrating adenosine beyond this point does not result in further hyperaemia,^20^ even though an increase in the rate of SSO can be observed.^21^ In accordance with this, only two patients in our study showed a reduced MFR, while one of those should be considered a true non-responder.

## New Knowledge Gained

SSO in PET MPI, as derived from SAR, corresponds with SSO in CMR and can be used as a marker for adequate adenosine response.

## Limitations

The low patient number may be perceived as a limitation of our study. However, as a pilot study used for the initial validation of SAR in selected patients undergoing hybrid PET/MR MPI, we believe that it allows drawing the above conclusions. Furthermore, we did not assess the predictive value of SAR to identify non-responders as only two patients had an impaired MFR. The patient population, consisting of both patients with coronary artery disease, as well as myocarditis may not be representative of patients undergoing routine PET MPI, so the applicability of the results should be verified in further studies, also including patients not abstaining from caffeine. It should be also noted that while the results of our study may be applicable to patients undergoing dipyridamole stress, the splenic switch-off sign is not observed after administration of regadenoson due to its selective receptor affinity or during dobutamine stress.^7^,^9^ Furthermore, since this study demonstrated the feasibility of detecting SSO in PET MPI as defined by visual SSO in CMR, further assessment of this sign using invasive coronary angiography and coronary computed angiography as an anatomical correlate is needed. Finally, our binary system of categorizing patients as responders and non-responders might be an over-simplification as a more graded adenosine response is suggested by some studies, depending on receptor density and responsiveness of adenosine receptors, as well as the distribution of SIR and activity ratios in our study suggest a graded response of splenic perfusion to adenosine stress.

## Conclusion

Similarly to CMR, SSO can be detected in nitrogen-13 ammonia PET MPI. This might help distinguish adenosine non-responders from patients with truly impaired MFR due to microvascular dysfunction or multivessel CAD.

## Abbreviations

CAD: Coronary artery disease
CMR: Cardiac magnetic resonance
MBF: Myocardial blood flow
MFR: Myocardial flow reserve
MPI: Myocardial perfusion imaging
PET: Positron emission tomography
PET/MR: Positron emission tomography/magnetic resonance
SAR: Splenic activity ratio
SIR: Splenic intensity ratio
SSO: Splenic switch-off
